# Choosing an appropriate glomerular filtration rate estimating equation: role of body mass index

**DOI:** 10.1186/s12882-021-02395-x

**Published:** 2021-05-25

**Authors:** Jiayong Li, Xiang Xu, Jialing Luo, Wenjing Chen, Man Yang, Ling Wang, Nan Zhu, Weijie Yuan, Lijie Gu

**Affiliations:** 1 Clinical Laboratory Medicine Center, Shanghai General Hospital, Shanghai Jiao Tong University School of Medicine, Shanghai, 200080 China; 2 Department of Nephrology, Shanghai General Hospital, Shanghai Jiao Tong University School of Medicine, Shanghai, 200080 China

**Keywords:** Body mass index, Estimated glomerular filtration rate, Chronic kidney disease

## Abstract

**Background:**

We aimed to investigate the accuracy of different equations in evaluating estimated glomerular filtration rate (eGFR) in a Chinese population with different BMI levels.

**Methods:**

A total of 837 Chinese patients were enrolled, and the eGFRs were calculated by three Chronic Kidney Disease Epidemiology Collaboration (CKD-EPI) equations, three full-age spectrum (FAS) equations and two Modification of Diet in Renal Disease (MDRD) equations. Results of measured GFR (mGFR) by the 99Tcm-diathylenetriamine pentaacetic acid (99Tcm-DTPA) renal dynamic imaging method were the reference standards. According to BMI distribution, the patients were divided into three intervals: below 25th(BMI_P25_), 25th to 75th(BMI_P25–75_) and over 75th percentiles (BMI_P75_).

**Results:**

The medium BMI of the three BMI intervals were 20.9, 24.8 and 28.9 kg/m^2^, respectively. All deviations from mGFR (eGFR) were correlated with BMI (*p* < 0.05). The percentage of cases in which eGFR was within mGFR ±30% (P30) was used to represent the accuracy of each equation. Overall, eGFR_FAS_Cr_CysC_ and eGFREPI_Cr_2009 performed similarly, showing the best agreement with mGFR among the eight equations in Bland-Altman analysis (biases: 4.1 and − 4.2 mL/min/1.73m^2^, respectively). In BMI_P25_ interval, eGFR_FAS_Cr_ got − 0.7 of the biases with 74.2% of P30, the kappa value was 0.422 in classification of CKD stages and the AUC_60_ was 0.928 in predicting renal insufficiency, and eGFREPI_Cr_2009 got 2.3 of the biases with 71.8% of P30, the kappa value was 0.418 in classification of CKD stages and the AUC_60_ was 0.920 in predicting renal insufficiency. In BMI_P25–75_ interval, the bias of eGFR_FAS_Cr_CysC_ was 4.0 with 85.0% of P30, the kappa value was 0.501 and the AUC_60_ was 0.941, and eGFR_FAS_Cr_CysC_ showed balanced recognition ability of each stage of CKD (62.3, 63.7, 68.0, 71.4 and 83.3% respectively). In BMI_P75_ interval, the bias of eGFR_EPI_Cr_CysC_2012_ was 3.8 with 78.9% of P30, the kappa value was 0.484 the AUC_60_ was 0.919, and eGFR_EPI_Cr_CysC_2012_ equation showed balanced and accurate recognition ability of each stage (60.5, 60.0, 71.4, 57.1 and 100% respectively). In BMI_P75_ interval, the bias of eGFR_FAS_Cr_CysC_ was − 1.8 with 78.5% of P30, the kappa value was 0.485, the AUC_60_ was 0.922. However, the recognition ability of each stage of eGFR_FAS_Cr_CysC_ eq. (71.1, 61.2, 70.0, 42.9 and 50.0% respectively) was not as good as GFR_EPI_Cr_CysC_2012_ equation.

**Conclusion:**

For a Chinese population, we tend to recommend choosing eGFR_FAS_Cr_ and eGFR_EPI_Cr_2009_ when BMI was around 20.9, eGFR_FAS_Cr_CysC_ when BMI was near 24.8, and eGFR_EPI_Cr_CysC_2012_ when BMI was about 28.9.

**Supplementary Information:**

The online version contains supplementary material available at 10.1186/s12882-021-02395-x.

## Introduction

Chronic kidney disease (CKD) is defined as a reduced glomerular filtration rate (GFR), increased urinary albumin excretion, or both, and has been recognized as an increasing public health issue worldwide [1]. Rising prevalence, poor outcomes, and high costs of CKD have led to considerable social and economic burdens in both developed and developing countries. Prevalence of CKD is estimated to be 8–16% worldwide [2]. In 2017, there were 132.3 million [95% confidence interval (95% CI) 121.8 to 143.7] people were diagnosed as CKD in China [3]. Therefore, the early prevention and accurate detection of CKD are particularly important.

Ideally, GFR should be measured. Measured (m) GFR gives an accurate assessment of kidney function and avoids confounding by interactions with variables, such as age or weight. Tc-99 m DTPA renal dynamic scintigraphy is a useful tool for clinicians in assessing renal function [4]. Because of the complicated process and nuclear pollution of above method, estimated GFR (eGFR) was considered as a convenient and no-invasive means which had been widely used in clinical diagnosis and treatment.

Many eGFR equations are based on the creatinine or/and cystatin C concentrations in serum. However, multiple factors such as muscle mass, weight, race, sex, gender and other individual differences affect the levels of serum creatinine [5]. Performance of Modification of Diet in Renal Disease (MDRD) and Chronic Kidney Disease Epidemiology Collaboration (CKD-EPI) equations remains suboptimal for estimating GFR in obese populations [6, 7]. Serum cystatin C also has the disadvantage in obesity population. Enlarged adipose tissues lead to elevation of serum cystatin C [8]. In fact, overweight and obesity account for a large proportion in CKD, while the muscle percentage is not synchronized with body weight. Therefore, the accuracy of eGFR assessments is affected by irregular fluctuation in creatinine and cystatin C.

How to choose an appropriate eGFR equation which can estimate renal function accurately? We used the body mass index (BMI) as the breakthrough point. There are many researches on the comparison of different eGFR equations, but still lack of researches on which special equation should be recommended in certain BMI range. In the present study, we assessed the accuracy of eight eGFR equations [CKD-EPI cr_2009 (eGFR_EPI_Cr_2009_) [9], CKD-EPI cys_2012 (eGFR_EPI_CysC_2012_) [1], CKD-EPI cr_cys_2012 (eGFR_EPI_Cr_CysC_2012_) [1, 10]], three full age spectrum (FAS) equations (eGFR_FAS _Cr_, eGFR_FAS _CysC_, and eGFR_FAS _Cr_CysC_) [11], abbreviated_MDRD (eGFR_a_MDRD_) [12], and Chinese_MDRD (eGFR_c_MDRD_) [13] compared with GFR measurement using 99Tcm-DTPA scintigraphy. Our research aimed to identify which equation performed better at estimating GFR and ideally predicting the CKD stage in the corresponding BMI interval, and finally, provide credible eGFR in certain BMI intervals to the clinicians.

## Methods

### Participants

A total of 904 patients who underwent GFR measurement using 99Tcm-diathylenetriamine pentaacetic acid (99Tcm-DTPA) scintigraphy from January 2016 to September 2017 in Shanghai General Hospital, were observed. Exclusion criteria included amputation, pregnant women, obstructive nephropathy, solitary kidney or a single kidney, urinary tract infection, acute kidney injury, any history of malignancy or kidney surgery, hyperthyroidism, use of antibacterial agents within 2 weeks, and malignant hypertension. Finally, a total of 837 patients were enrolled in this study. General characteristics were included such as sex, age, body mass index (BMI), serum creatinine, serum cystatin C, measured GFR (mGFR) and the situation of basic diseases. BMI was calculated following the equation: BMI (Kg/m^2^) = weight (kg) /height^2^ (m). Three intervals were divided based on BMI percentiles, percentile 25% (BMI_P25_), percentile 25% ~ 75% (BMI_P25–75_) and percentile 75% (BMI_P75_). Research has been conducted in accordance with the Declaration of Helsinki and was approved by the Ethics Committee of Shanghai General Hospital. Written informed consent was obtained from all participants. All methods were carried out in accordance with the relevant guidelines and regulations.

### Measurement of reference GFR (mGFR)

The mGFR was measured by gate’s method of radionuclide renal dynamic imaging. The instrument used Siemens Excel Evo SPECT which equipped with low energy and high resolution parallel hole collimator, energy peak 140 keV, window width ± 20%. 99TcmDTPA (radiochemical purity, > 95%; percentage of 99TcmDTPA bound to plasma protein, < 5%) was provided by Shanghai Atom Kexing Pharmaceutical Co., Ltd., China. Determined the mGFR by gate’s method.

### Definition of renal insufficiency and CKD classification

The definition of renal insufficiency and CKD classification were referred to the 2012 KDIGO clinical practice guideline [1]. Renal insufficiency was defined as mGFR < 60 mL/min/1.73 m^2^. CKD was classified into five stages based on the mGFR values as follows: stage 1, mGFR ≥90 mL/min/1.73 m^2^; stage 2, 60 mL/min/1.73 m^2^ ≤ mGFR < 90 mL/min/1.73 m^2^; stage 3, 30 mL/min/1.73 m^2^ ≤ mGFR < 60 mL/min/1.73 m^2^; stage 4, 15 mL/min/1.73 m^2^ ≤ mGFR < 30 mL/min/1.73 m^2^; stage 5, mGFR < 15 mL/min/1.73 m^2^.

### Measurement of serum creatinine (Scr) and cystatin C (CysC) levels and GFR-estimating equations

Blood samples were obtained after the patients had fasted for 12 h. Both Scr and CysC were measured by an automatic biochemical autoanalyzer (Cobas 8000; Roche Products Ltd. Basel, Switzerland), used original matching assay kit (Roche Diagnostics, Mannheim, Germany). Based on the Scr, eGFR was calculated by CKD-EPI Cr_2009 (eGFR_EPI_Cr_2009_) [9], FAS Cr (eGFR_FAS_Cr)_ [11], abbreviated _MDRD (eGFR_a_MDRD_) [12], and Chinese _MDRD (eGFR_c_MDRD_) [13]. Based on the CysC, eGFRs was calculated by CKD-EPI CysC_2012 (eGFR_EPI_CysC_2012_) [1] and FAS CysC (eGFR_FAS _CysC)_ [11]. Based on both SCr and CysC, eGFR was calculated by CKD-EPI Cr_CysC_2012 (eGFR_EPI_Cr_CysC_2012_) [10] and FAS Cr_CysC (eGFR_FAS _Cr_CysC_) [11].

The equations used in the study population (with no correction for race and ethnicity) were the following (SCr indicates serum creatinine):

1. CKD-EPI Cr_2009 equation:
  $$ \mathrm{Female},\mathrm{SCr}\le 61.88\upmu \mathrm{mol}/\mathrm{L}:\mathrm{eGFR}=144\times {\left(\mathrm{SCr}/61.88\right)}^{-0.329}\times {0.993}^{\mathrm{age}}\times \left(1.159\mathrm{ifblack}\right)\mathrm{Female},\mathrm{SCr}>61.88\upmu \mathrm{mol}/\mathrm{L}:\mathrm{eGFR}=144\times {\left(\mathrm{SCr}/61.88\right)}^{-1.209}\times {0.993}^{\mathrm{age}}\times \left(1.159\mathrm{ifblack}\right)\mathrm{Male},\mathrm{SCr}\le 79.56\upmu \mathrm{mol}/\mathrm{L}:\mathrm{eGFR}=141\times {\left(\mathrm{SCr}/79.56\right)}^{-0.411}\times {0.993}^{\mathrm{age}}\times \left(1.159\mathrm{ifblack}\right)\mathrm{Male},\mathrm{SCr}>79.56\upmu \mathrm{mol}/\mathrm{L}:\mathrm{eGFR}=141\times {\left(\mathrm{SCr}/79.56\right]}^{-1.209}\times {0.993}^{\mathrm{age}}\times \left(1.159\mathrm{ifblack}\right) $$
2. CKD-EPI CysC_2012 equation:
  $$ \mathrm{Female},\mathrm{SCr}\le 61.88\upmu \mathrm{mol}/\mathrm{L}\ \mathrm{and}\ \mathrm{SCys}\le 0.8\mathrm{mg}/\mathrm{dL}:\mathrm{eGFR}=130\times {\left(\mathrm{Scr}/61.88\right)}^{-0.248}\times {\left(\mathrm{Scyst}/0.8\right)}^{-0.375}\times {0.995}^{\mathrm{age}}\times \left(1.08\ \mathrm{if}\ \mathrm{black}\right)\mathrm{Female},\mathrm{SCr}\le 61.88\upmu \mathrm{mol}/\mathrm{L}\ \mathrm{and}\ \mathrm{SCys}>0.8\ \mathrm{mg}/\mathrm{dL}:\mathrm{eGFR}=130\times {\left(\mathrm{Scr}/61.88\right)}^{-0.248}\times {\left(\mathrm{Scyst}/0.8\right)}^{-0.711}\times {0.995}^{\mathrm{age}}\times \left(1.08\ \mathrm{if}\ \mathrm{black}\right)\mathrm{Female},\mathrm{SCr}>61.88\upmu \mathrm{mol}/\mathrm{L}\ \mathrm{and}\ \mathrm{SCys}\le 0.8\ \mathrm{mg}/\mathrm{dL}:\mathrm{eGFR}=130\times {\left(\mathrm{Scr}/61.88\right)}^{-0.601}\times {\left(\mathrm{Scyst}/0.8\right)}^{-0.375}\times {0.995}^{\mathrm{age}}\times \left(1.08\ \mathrm{if}\ \mathrm{black}\right)\mathrm{Female},\mathrm{SCr}>61.88\upmu \mathrm{mol}/\mathrm{L}\ \mathrm{and}\ \mathrm{SCys}\ge 0.8\mathrm{mg}/\mathrm{dL}:\mathrm{eGFR}=130\times {\left(\mathrm{Scr}/61.88\right)}^{-0.601}\times {\left(\mathrm{Scyst}/0.8\right)}^{-0.711}\times {0.995}^{\mathrm{age}}\times \left(1.08\ \mathrm{if}\ \mathrm{black}\right)\mathrm{Male},\mathrm{SCr}\le 79.56\upmu \mathrm{mol}/\mathrm{L}\ \mathrm{and}\ \mathrm{SCys}\le 0.8\ \mathrm{mg}/\mathrm{dL}:\mathrm{eGFR}=135\times {\left(\mathrm{Scr}/79.56\right)}^{-0.207}\times {\left(\mathrm{Scyst}/0.8\right)}^{-0.375}\times {0.995}^{\mathrm{age}}\times \left(1.08\ \mathrm{if}\ \mathrm{black}\right)\mathrm{Male},\mathrm{SCr}\le 79.56\upmu \mathrm{mol}/\mathrm{L}\ \mathrm{and}\ \mathrm{SCys}>0.8\mathrm{mg}/\mathrm{dL}:\mathrm{eGFR}=135\times {\left(\mathrm{Scr}/79.56\right)}^{-0.207}\times {\left(\mathrm{Scyst}/0.8\right)}^{-0.711}\times {0.995}^{\mathrm{age}}\times \left(1.08\ \mathrm{if}\ \mathrm{black}\right)\mathrm{Male},\mathrm{SCr}>79.56\upmu \mathrm{mol}/\mathrm{L}\ \mathrm{and}\ \mathrm{SCys}\le 0.8\ \mathrm{mg}/\mathrm{dL}:\mathrm{eGFR}=135\times {\left(\mathrm{Scr}/79.56\right)}^{-0.601}\times {\left(\mathrm{Scyst}/0.8\right)}^{-0.375}\times {0.995}^{\mathrm{age}}\times \left(1.08\ \mathrm{if}\ \mathrm{black}\right)\mathrm{Male},\mathrm{SCr}>79.56\upmu \mathrm{mol}/\mathrm{L}\ \mathrm{and}\ \mathrm{SCys}>0.8\ \mathrm{mg}/\mathrm{dL}:\mathrm{eGFR}=135\times {\left(\mathrm{Scr}/79.56\right)}^{-0.601}\times {\left(\mathrm{Scyst}/0.8\right)}^{-0.711}\times {0.995}^{\mathrm{age}}\times \left(1.08\ \mathrm{if}\ \mathrm{black}\right) $$
3. CKD-EPI CysC_2012 equation:
  $$ \mathrm{SCys}\le 0.8\mathrm{mg}/\mathrm{L}:\mathrm{eGFR}=133\times {\left(\mathrm{Scys}\mathrm{t}/0.8\right)}^{-0.499}\times {0.996}^{\mathrm{age}}\left(\times 0.932\ \mathrm{if}\ \mathrm{female}\right)\mathrm{SCys}>0.8\mathrm{mg}/\mathrm{L}:\mathrm{eGFR}=133\times {\left(\mathrm{Scys}/0.8\right)}^{-1.328}\times {0.996}^{\mathrm{age}}\left(\times 0.932\ \mathrm{if}\ \mathrm{female}\right) $$
4. FAS Cr equation:
  $$ \mathrm{eGFR}=107.3/\left(\mathrm{SCr}/\mathrm{QCys}\right)\times \left[{0.988}^{\left(\mathrm{age}-40\right)},\mathrm{when}\ \mathrm{age}>40\ \mathrm{years}\right]\left(\mathrm{female}:\mathrm{QScr}=0.70\ \mathrm{mg}/\mathrm{dl};\mathrm{male}:\mathrm{QScr}=0.90\ \mathrm{mg}/\mathrm{dl}\right); $$
5. FAS CysC equation:
  $$ \mathrm{eGFR}=107.3/\left(\mathrm{SCys}/\mathrm{QCys}\right]\times \left[{0.988}^{\left(\mathrm{age}-40\right)}\ \mathrm{when}\ \mathrm{age}>40\ \mathrm{years}\right]\left(\mathrm{age}<70\ \mathrm{years}\ \mathrm{old}:\mathrm{QCys}=0.82\ \mathrm{mg}/\mathrm{l};\mathrm{age}\ge 70\ \mathrm{years}\ \mathrm{old}:\mathrm{QCys}=0.95\ \mathrm{mg}/\mathrm{l}\right) $$
6. FAS Cr-CysC equation:
  $$ \mathrm{eGFR}=107.3/\left[\upalpha \times \left(\mathrm{SCr}/\mathrm{QScr}\right)+\left(1-\upalpha \right)\times \left(\mathrm{SCys}/\mathrm{QCys}\right)\right]\times \left[{0.988}^{\left(\mathrm{age}-40\right)}\ \mathrm{when}\ \mathrm{age}>40\ \mathrm{years}\right]\left(\mathrm{female}:\mathrm{QScr}=0.70\ \mathrm{mg}/\mathrm{dl};\mathrm{male}:\mathrm{QScr}=0.90\ \mathrm{mg}/\mathrm{dl};\mathrm{age}<70\ \mathrm{years}\ \mathrm{old}:\mathrm{QCys}=0.82\ \mathrm{mg}/\mathrm{l};\mathrm{age}\ge 70\ \mathrm{years}\ \mathrm{old}:\mathrm{QCys}=0.95\ \mathrm{mg}/\mathrm{l};\upalpha =0.5\right) $$
7. abbreviated_MDRD equation:
  $$ \mathrm{eGFR}=175\times \mathrm{SCr}\ {\left(\upmu \mathrm{mol}/\mathrm{L}\times 0.0011312\right)}^{-1.154}\times \mathrm{age}\ {\left(\mathrm{years}\right)}^{-0.203}\times \left(0.742,\mathrm{if}\ \mathrm{female}\right)\times \left(1.212,\mathrm{if}\ \mathrm{black}\right) $$
8. Chinese _MDRD equation:
  $$ \mathrm{eGFR}=175\times \mathrm{SCr}\ {\left(\upmu \mathrm{mol}/\mathrm{L}\times 0.0011312\right)}^{-1.234}\times \mathrm{age}\ {\left(\mathrm{years}\right)}^{-0.179}\times \left(0.79,\mathrm{if}\ \mathrm{female}\right) $$

### Statistical analysis

Statistical analyses were performed using SPSS version 22.0 for Windows (SPSS Inc., Chicago, USA) and Medcalc 11.4 for windows. Kolmogorov-Smirnov test (K-S) was used to test the normality of variables [14]. Continuous variables were presented as the means ± standard deviation and were analyzed using unpaired Student’s t-tests. Nonnormally distributed variables were presented as medians with corresponding 25th and 75th percentiles (interquartile ranges) and compared using the Mann–Whitney U test [15]. Wilcoxon test was used to compare the differences of the deviation from mGFR (△eGFR, which is mGFR minus eGFR) by these eight eGFRs when in different BMI interval. Plotting scatter diagrams were used to observe the trend of each △eGFR when in different BMI state. Partial correlation analysis was used to evaluate correlations between △eGFR and BMI. Bland-Altman analysis [16] was used to determine the agreement between the mGFR and eGFR values, similar to the study by Chi et al [17], which were calculated by different equations. The percentage of cases in which eGFR was within mGFR ±30% (P30) was used to represent the accuracy of each equation. Kappa statistics were used to evaluate the agreement between stage classification from the mGFR values and from the eGFR values calculated by different equations, with the following interpretations: slight agreement (0–0.20), fair agreement (0.21–0.40), moderate agreement (0.41–0.60), substantial agreement (0.61–0.80), and almost perfect or perfect agreement (0.81–1.0) [18]. The receiver operating characteristic (ROC) curve was used to determine the diagnostic power at predicting the renal insufficiency (ROC_60_) by the eight different equations, with the results reported as the areas under the ROC curve (AUC_60_), sensitivity, and specificity [19]. Differences with *P* < 0.05 were considered statistically significant.

## Results

### Overview of the entire study population

The demographic and clinical features of the participants included in the analysis are listed in Table 1. The medium BMI was 24.8 Kg/m^2^ which 25.1 Kg/m^2^ for male, and 24.2 for female. According to the percentile of BMI, it was divided into three intervals, < 25% (BMI_P25_), 25% ~ 75% (BMI_P25–75_) and > 75% (BMI_P75_), respectively. The average BMI was 20.9 Kg/m^2^ in BMI_P25,_ 24.8 Kg/m^2^ in BMI_P25–75,_ and 28.9 Kg/m^2^ in BMI_P75_ respectively. Among all the patients, 66.8% had diabetes and 64.5% had hypertension, and 71.6% had atherosclerosis which was the most common diagnosis. The average mGFR was 71.4 ± 28.1 mL/min/1.73 m^2^, while the average eGFR varied according to different calculation formulas, ranging from 60.2(44.9, 74.9)mL/min/1.73 m^2^ to 88.5 ± 49.1 mL/min/1.73 m^2^.

**Table 1:** Baseline characteristics

Variable	Total	Male(***n*** = 505)	Female(***n*** = 332)
Age, years	60 (52, 69)	59 (51, 67)	62 (55, 71) *
Height, cm	167 (160.0, 172.3)	170 (167, 175)	160 (155, 163) *
Weight, kg	69 (60, 77)	74 (66, 81)	61 (54, 69) *
Body surface area (BSA), m^2^	1.81 (1.67, 1.95)	1.90 (1.80, 2.01)	1.66 (1.56, 1.77) *
Body mass index (BMI), Kg/m^2^	24.8 (22.7, 27.3)	25.1(23.1, 27.4)	24.2(21.6, 26.8) *
BMI_P25_, Kg/m^2^ (*n* = 209)	20.9 (19.6, 21.9)		
BMI_P25–75_, Kg/m^2^ (*n* = 419)	24.8 (23.7, 25.9)		
BMI_P75_, Kg/m^2^ (n = 209)	28.9 (28.0, 30.6)		
Serum creatinine (sCr), μmol/L	79 (61, 113.3)	86 (68, 120)	66 (51, 100) *
Serum uric acids (sUA), μmol/L	360 (289, 435)	380 (311, 449)	323 (268, 421) *
Serum urea (sUrea), mmol/L	6.3 (5.0, 8.6)	6.5 (5.2, 8.6)	6.1 (4.8, 8.8) ^△^
Serum cystatin C (sCysc), mg/L	1.17 (0.97, 1.53)	1.18 (0.99, 1.53)	1.16 (0.94, 1.58) ^△^
Urinary albumin creatinine ratio (ACR), μg/mg	64.4 (18.3, 458.5)	73.8 (15.7, 522.8)	60.2 (23.9, 406.1) ^△^
ACR ≥ 30 μg/mg rate, %	64.03	62.80	66.10^△^
Diabetes	559 (66.8%)	373 (73.9%)	186 (56.0%)*
Hypertension	540 (64.5%)	325 (64.4%)	215 (42.6%)^△^
coronary heart disease	165 (19.7%)	102 (20.2%)	63 (12.5%)^△^
atherosclerosis	599 (71.6%)	358 (70.9%)	241 (47.7%)^△^
mGFR, ml·min −1·1.73 m 2	71.4 ± 28.1	69.8 ± 27.3	73.8 ± 29.2^▲^
distribution in each CKD stage			
CKD1	221 (26.4%)	123 (24.4%)	98 (29.5%)^△^
CKD2	338 (40.4%)	214 (42.4%)	124 (37.4%)^△^
CKD3	212 (25.3%)	130 (25.7%)	82 (24.7%)^△^
CKD4	54 (6.5%)	28 (5.5%)	26 (7.8%)^△^
CKD5	12 (1.4%)	10 (2.0%)	2 (0.6%)^△^
eGFR			
eGFR_a_MDRD_	75.3(47.2, 105.1)	78.6(55.8, 104.7)^★^	78.2(48.0, 110.0)^★△^
eGFR_c_MDRD_	88.5 ± 49.1	87.9 ± 44.7^★^	98.4 ± 54.4^★▲^
eGFR_EPI_Cr_2009_	81.9 (52.2, 93.3)	82.5 (55.5, 101.2) ^★^	80.4 (48.5, 97.8) ^★△^
eGFR_EPI_Cr_CysC_2012_	63.3 (42.0, 81.2)	63.5 (43.6,79.8) ^★^	62.2 (39.6, 82.2) ^★△^
eGFR_EPI_CysC_2012_	62.8 (43.4, 82.3)	62.5 (44.1, 80.3) ^★^	62.8 (41.2, 84.5) ^★△^
eGFR_FAS_Cr_	77.7(51.6, 104.0)^★^	78.8 ± 36.7^★^	74.1(48.1, 104.6)^□△^
eGFR_FAS_CysC_	60.2(44.9, 74.9)^★^	60.8(45.9, 73.9)^★^	61.4 ± 24.8^★△^
eGFR_FASCr_CysC_	66.8(47.8, 85.6)^★^	67.9 ± 28.3 ^★^	66.0(46.3, 87.3) ^★△^

^△^*P* > 0.05, ^▲^*P* < 0.05, *P < 0.01, compared with male; ^★^*P* < 0.01, ^□^*P* > 0.05, compared with mGFR

### Relation between BMI and △eGFR based on different formulas

**Fig. 1:**
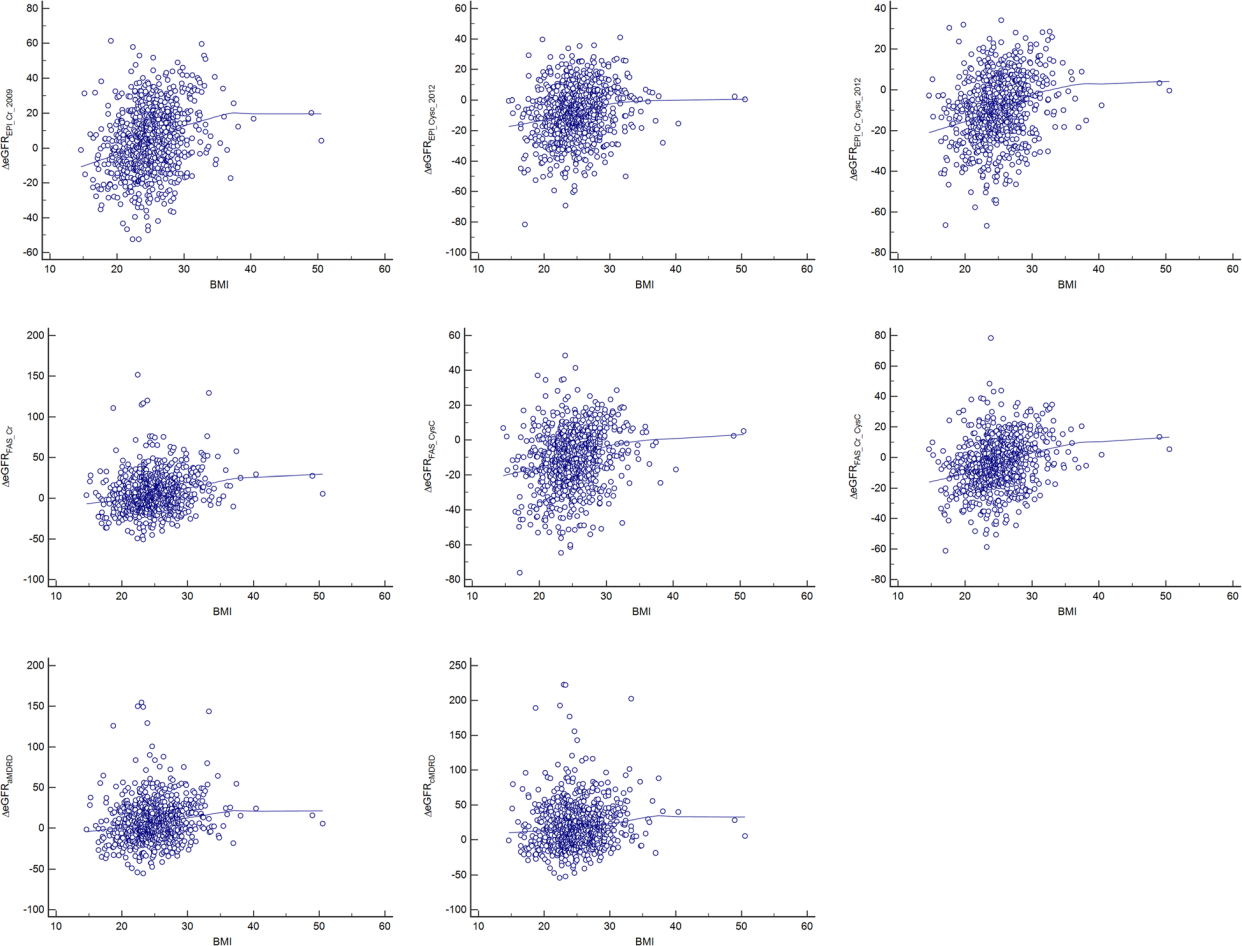
Plot scatter diagrams of △eGFR based on different formulas with the increase of BMI

The correlation between △eGFR based on different formulas and BMI was shown by plotting scatter diagrams of △eGFR based on different formulas with the increase of BMI (Fig. 1). With the increase of BMI, trends of △eGFR differed with diverse formulas. Partial correlation coefficient was shown in Table 2, which was statistically significant (*p* = 0.012 for △eGFR_c_MDRD_ while the rest *p* < 0.001).

**Table 2:** Partial correlation analysis between △eGFR based on different formulas and BMI

	△eGFR_EPI_Cr_2009_	△eGFR_EPI_CysC_2012_	△eGFR_EPI_Cr_CysC_2012_	△eGFR_FAS_Cr_	△eGFR_FAS_CysC_	△eGFR_FAS_Cr_CysC_	△eGFR_a_MDRD_	△eGFR_c_MDRD_
r	0.264	0.197	0.260	0.210	0.222	0.267	0.140	0.087
*p* value	< 0.0001	< 0.0001	< 0.0001	< 0.0001	< 0.0001	< 0.0001	0.000	0.012

The comparison of △eGFR among different BMI groups was shown in Table 3. Delta eGFR_EPI_Cr_2009_, △eGFR_EPI_CysC_2012_, △eGFR_EPI_Cr_CysC_2012_, △eGFR_FAS___Cr_, △eGFR_FAS_CysC_ and △eGFR_FAS_Cr_CysC_ showed significant differences in different BMI intervals (*p* = 0.030, 0.010, 0.000, 0.0029, 0.000 and 0.001 respectively). While △eGFR_a_MDRD_ and △eGFR_c_MDRD_ had no significant difference in different BMI intervals (*p* = 0.234 and 0.522, respectively).

**Table 3:** Comparison of △eGFR among different BMI groups

	△eGFR_EPI_Cr_2009_	△eGFR_EPI_CysC_2012_	△eGFR_EPI_Cr_CysC_2012_	△eGFR_FAS_Cr_	△eGFR_FAS_CysC_	△eGFR_FAS_Cr_CysC_	△eGFR_a_MDRD_	△eGFR_c_MDRD_
BMI < 25%	10.8(5.6, 21.0)	15.3(8.0, 26.9)	15.1(8.4, 25.5)	12.9(6.2, 21.8)	16.7(8.4, 28.6)	12.3(6.5, 22.1)	15.8(6.8, 25.0)	20.4(9.4, 37.5)
BMI 25–75%	12.1(6.0, 20.0)	12.2(6.2, 19.4)	11.7(6.1, 19.0)	11.9(6.0, 23.6)	12.1(5.2, 21.5)	9.1 (4.6, 16.6)	13.7(6.1, 25.7)	19.4(8.8, 38.5)
BMI > 75%	15.5(7.0, 24.8)	10.2(5.0, 18.9)	9.3(4.6, 18.6)	15.5(7.3, 25.2)	9.6 (4.4, 17.5)	10.3(4.7, 19.0)	16.3(7.4, 27.6)	21.9(10.2, 39.0)
p value	0.030	0.010	0.000	0.029	0.000	0.001	0.234	0.522

### Consistency of eGFRs compared with mGFR

**Fig. 2:**
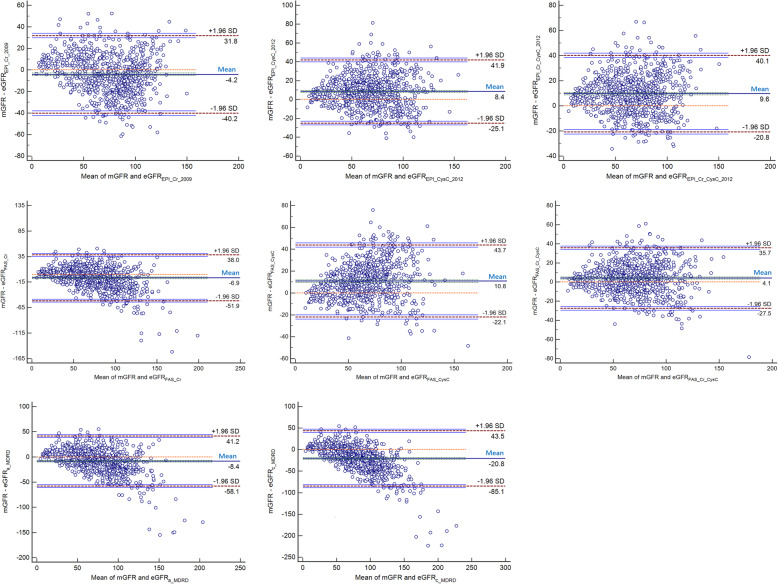
Bland-Altman plots of the mGFR and eGFR (mL/min/1.73 m^2^)

The consistency between the eGFR based on different formulas and the mGFR was analyzed by Bland-Altman plots (Fig. 2, Table 4). The accuracy of each equation was represented by the percentage of cases in which eGFR was within the range of mGFR ±30% (P30). Compared with mGFR, biases of eGFR_FAS_Cr_CysC_ and eGFR_EPI_Cr_2009_ (4.1 and − 4.2, respectively) were much less than those of eGFR_FAS_Cr_ eGFR_EPI_CysC_2012_, eGFR_EPI_Cr_CysC_2012_, eGFR_FAS_CysC_, eGFR_a_MDRD_ and eGFR_c_MDRD_ (− 6.9, 8.4, 9.6, 10.8, − 8.4 and − 20.8, respectively). The accuracy of each eGFR was as follows: 81.5% for eGFR_FAS_Cr_CysC_, 74.1% for eGFR_EPI_Cr_CysC_2012_, 74.1% for eGFR_EPI_CysC_2012_, 73.4% for eGFR_FAS_CysC_, 70.1% for eGFR_EPI_Cr_2009_, 69.3% for eGFR_FAS_Cr_, 63.0%for eGFR_a_MDRD_ and 47.0% for eGFR_c_MDRD_.

**Table 4:** Comparison of bias and accuracy between eGFRs and mGFR in different BMI groups

BMI	Total		< 25%		25–75%		> 75%	
Equation	Bias (mL/min/1.73 m^2^) (△eGFR, 95% CI)	30% accuracy	Bias (mL/min/1.73 m^2^) (△eGFR, 95% CI)	30% accuracy	Bias (mL/min/1.73 m^2^) (△eGFR, 95% CI)	30% accuracy	Bias (mL/min/1.73 m^2^) (△eGFR, 95% CI)	30% accuracy
eGFR_EPI_Cr_2009_	−4.2(− 5.5 - -3.0)^△^	70.1%	2.3(−0.1–4.8**)**^□^	71.8%	−4.0(−5.8 - -2.5)^△^	74.7%	−11.1(−13.5 - -8.4)^△^	59.3%
eGFR_EPI_CysC_2012_	8.4(7.3–9.6)^△^	74.0%	13.1(10.7–15.7)^△^	67.5%	8.6(7.0–10.1)^△^	75.9%	3.3(1.2–5.5)^▲^	76.6%
eGFR_EPI_Cr_CysC_2012_	9.6(8.6–10.7)^△^	74.1%	15.2(13.1–17.3)^△^	64.1%	9.8(8.4–11.2)^△^	75.4%	3.8(1.7–5.8)^△^	78.9%
eGFR_FAS_Cr_	−6.9(−8.5 - -5.4)^△^	69.3%	−0.7(−3.8–2.5)^○^	74.2%	−7.1(−9.2 - -4.9)^△^	70.6%	−12.9(− 16.0 –− 9.9)^△^	61.7%
eGFR_FAS_CysC_	10.8(9.7–11.9)^△^	73.4%	15.9(13.5–18.3)^△^	64.1%	11.0(9.5–12.6)^△^	76.1%	5.3(3.2–7.4)^△^	77.0%
eGFR_FAS_Cr_CysC_	4.1(3.0–5.1)^△^	81.5%	9.9(7.8–12.1)^△^	77.5%	4.0(2.5–5.5)^△^	85.0%	−1.8(− 3.9–0.3)^●^	78.5%
eGFR_a_MDRD_	−8.4(− 10.2 - -6.7)^△^	63.0%	−3.7(− 7.3 - -0.1) ^▲^	64.6%	− 8.5(− 10.9 - -6.0)^△^	65.0%	−13.1(− 16.3 - -9.9)^△^	59.8%
eGFR_c_MDRD_	−20.8(−23.0 - -18.6)^△^	47.0%	−8.8(− 12.6 - -4.9)^△^	45.5%	−20.5(−23.9 - -17.5)^△^	51.1%	−24.8(−28.6 - -20.5)^△^	40.2%

△Compared with mGFR, *P* < 0.01;

▲Compared with mGFR, *P* < 0.05;

□Compared with mGFR, P = 0.061;

■Compared with mGFR, *P* = 0.401

○Compared with mGFR, P = 0.679;

●Compared with mGFR, P = 0.095;

However, the accuracy of different formulas varied in different BMI intervals. In the BMI_P25_ interval, the bias of eGFR_FAS_Cr_ was improved to − 0.7 mL/min/1.73 m^2^(*P* = 0.679) with 74.2% of P30. And the bias of eGFR_EPI_Cr_2009_ was 2.3 mL/min/1.73 m^2^ (*P* = 0.061) with 71.8% of P30. In the range of BMI_P25–75_, the bias of eGFR_FAS_Cr_CysC_ was 4.0 mL/min/1.73 m^2^ with 85.0% of P30, and the bias of eGFR_EPI_Cr_2009_ was 4.0 mL/min/1.73 m^2^ with 74.7% of P30, which were most consistent with mGFR. In the BMI_P75_ interval, the bias of eGFR_EPI_Cr_CysC_2012_ was 3.8 mL/min/1.73 m^2^ (*P* < 0.01) with 78.9% of P30 and the bias of eGFR_EPI_CysC_2012_ was 3.3 mL/min/1.73 m^2^ *(P* < 0.05*)* with 76.6% of P30. The bias of eGFR_FAS_Cr_CysC_ was − 1.8 mL/min/1.73 m^2^, but there was no statistical significance (*P* = 0.095). It was suggested that the consistency of eGFR compared with the mGFR was the best when eGFR calculated by eGFR_EPI_CysC_2012_ and eGFR_FAS_Cr_CysC_ formulas.

### Accuracy of eGFR in CKD staging in different BMI intervals

The kappa values of eGFR_EPI_Cr_2009_, eGFR_FAS_Cr_ and eGFR_a_MDRD_ were similar(0.418, 0.422 and 0.412 respectively), which were higher than that of other formulas when in BMI_P25_ interval (Supplemental Table 1). They showed high accuracy (84.4, 76.6 and 88.3%, respectively) in the identification of stage 1 CKD and moderate accuracy in the identification of stage 2 and 3 CKD. In BMI_P25–75_ interval, eGFR_FAS_Cr_CysC_ had highest kappa value (0.504), which was higher than eGFR_EPI_CysC_2012_ (0.431), eGFR_FAS_Cr_(0.415) and eGFR_EPI_Cr_CysC_2012_ (0.415). The eGFR_FAS_Cr_CysC_ showed a better accuracy in the identification of stage 2 and 3 CKD (63.7 and 68.0% respectively) (Supplemental Table 2). In the BMI_P75_ interval, eGFREPI_Cr_CysC_2012 was found to be the best, with a kappa value of 0.484, showing balanced and accurate recognition ability of each stage (60.5, 60.0, 71.4, 57.1 and 100% respectively) (Supplemental Table 3). However, the recognition ability of each CKD stage of FAS_Cr_CysC eq. (71.1, 61.2, 70.0, 42.9 and 50.0% respectively) was not as good as EPI_Cr_CysC_2012 equation.

### Diagnostic performance of each eGFR equation for predicting renal insufficiency in different BMI intervals

**Fig. 3:**
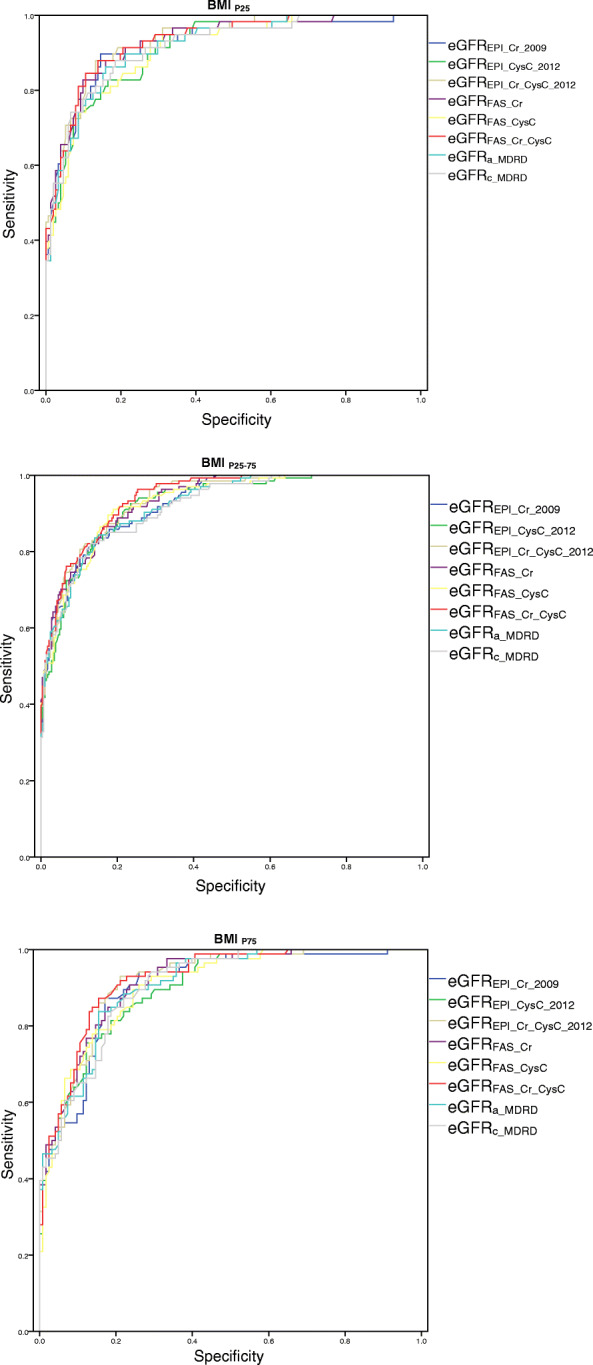
Receiver operating characteristic curve of eGFRs in different BMI intervals

The diagnostic performance for predicting renal insufficiency based on each eGFR equation in three BMI intervals was summarized and showed in Supplemental Table 4, 5 and 6 and Fig.3. In the BMI_P25_ interval, with a sensitivity of 89.7% and a specificity of 84.1%, at a cut-off point of 67.1 mL/min/1.73 m^2^, eGFREPI_Cr_2009 got an AUC_60_ of 0.920 which had no significant difference compared with other equations (*p* > 0.05), suggesting appropriate diagnostic ability for predicting renal insufficiency. In BMI_P25_ interval, eGFR_FAS_Cr_ had similar performance with a sensitivity of 79.3%, specificity of 88.1%, and an AUC_60_ of 0.928 at a cut-off point of 56.6 mL/min/1.73 m^2^.

In BMI_P25–75_ interval, the cut-off point of eGFR_FAS_Cr_CysC_ was 62.9 mL/min/1.73 m^2^, with an AUC_60_ of 0.941 which had no significant difference compared with other equations (except eGFR_FAS_CysC_, *p* = 0.021). When the cut-off values of eGFREPI_CysC_2012 and eGFREPI_Cr_CysC_2012 were revised to 60.1 and 60.2 mL/min/ 1.73 m^2^ respectively, the sensitivity was increased to 90.3 and 91.8% respectively, but the specificity was decreased to 78.9 and 77.2% respectively. Looking back at eGFR_FAS_Cr_CysC_, when cut-off point of eGFR_FAS_Cr_CysC_ was 62.9 mL/min/1.73 m^2^, the sensitivity was 92.5% and the specificity was 78.6%, while after revising cut-off value to 60.0 mL/min/1.73 m^2^, the sensitivity was 87.3% and the specificity was 82.0%, indicating that the diagnostic performance for predicting renal insufficiency was relatively stable. In BMI_P75_ interval, the optimal cut-off point of eGFR_EPI_Cr_CysC_2012_ for predicting renal insufficiency was 60.5 mL/min/1.73 m^2^, with an ideal sensitivity of 90.7%, a specificity of 80.5%, and an AUC_60_ of 0.919 (*P* < 0.05 vs. eGFR_EPI_CysC_2012_), highlighted itself. The optimal cut-off point of eGFR_FAS_Cr_CysC_ for predicting renal insufficiency was 61.5 mL/min/1.73 m^2^, with a sensitivity of 87.2%, a specificity of 84.6%, and an AUC_60_ of 0.922 (*P* < 0.05 vs. eGFR_FAS_CysC_). It suggested that eGFR_EPI_Cr_CysC_2012_ and eGFR_FAS_Cr_CysC_ had the strongest ability to predict renal insufficiency in BMI_P75_ interval.

## Discussion

There is high disease burden of CKD in China [2]. The global increase in this disease is mainly driven by the increase in the prevalence of diabetes mellitus, hypertension, obesity, and aging. To make matters worse, the risk of death gradually increases with the deterioration of CKD [20]. Therefore, screening, diagnosis, and staging CKD early as well as accurately are more and more important. Estimating GFR accurately is crucial for clinical practice, research, and public health. Although Tc-99 m DTPA renal dynamic scintigraphy is a useful tool for clinicians in assessing renal function, this method cannot be regularly used in clinical practice. On the contrary, GFR estimated from equations is a convenient approach to assess patients’ renal function. Due to the convenience of testing, it can be used as a method for large-scale cases screening.

Each eGFR equation is established by statistically processing of certain population data, so it always performs less well outside the cohort in which they were developed [21]. All methods for the estimation of GFR have limitations, so no equation can perform best in all populations. Obesity is associated with a risk of CKD and is highly prevalent among patients with CKD [22, 23]. In our study, the average BMI of the cases was 24.8, of which 25.1 for males and 24.2 for females. A large number of patients were overweight or obese. Therefore, it inspired us to consider the influence of BMI, which can partly reflect the difference of body. If we properly handled this influence, can we make the best use of each eGFR equation? There are few studies on the applicability of different eGFR equations in different BMI intervals. In this study, we evaluated the value of different eGFR formulas in different BMI intervals.

After being analyzed by Bland-Altman plots, biases of eGFR_FAS_Cr_CysC_ and eGFR_EPI_Cr_2009_ were much less than that of others on the whole, showing the best agreement with mGFR. In BMI_P25_ interval, eGFR_FAS_Cr_ and eGFR_EPI_Cr_2009_ formulas had optimal accuracy, excellent ability to classify CKD stages, and best diagnostic performance for predicting renal insufficiency. In BMI_P25–75_ interval, eGFR_FAS_Cr_CysC_ was the best one, with optimal accuracy and excellent ability in staging CKD2 and CKD3. In BMI_75_ interval, eGFR_EPI_Cr_CysC_2012_ equation showed excellent accuracy, stable identification power for CKD stages and the strongest ability to predict renal insufficiency. In BMI_75_ interval, the accuracy and ability to predict renal insufficiency of eGFR_FAS_Cr_CysC_ was similar to that of eGFR_EPI_Cr_CysC_2012_. However, eGFR_FAS_Cr_CysC_ was not as good as eGFRE_PI_Cr_CysC_2012_ equation in identifying CKD stages. We found Scr-cysC-based eGFR equations had superiority in evaluating eGFR compared to the Scr-based formulas in overweight or obese people.

It’s well known that SCr has limitations including its insensitivity to underlying changes in kidney function and the numerous non-kidney factors that are incompletely accounted for in equations to eGFR [24]. Although as an endogenous biomarker, concentration of cysC also can be affected by other non-renal determinants, such as obesity, thyroid disorders, diabetes, and inflammation, however, compared to SCr, cysC appears to be less affected by age, race, sex, muscle mass, or dietary intake [25, 26]. It is increasingly accepted to use the use equations based on cystatin C or combined creatinine and cystatin C [27]. In fact, kidney function assessment in obese patients is challenging. Nephron number does not change with weight gain, and the increase of GFR observed in obese patients reflects compensatory hyperfiltration of nephrons. This hyperfiltration in obese patients can become maladaptive and is largely unaccounted for in existing eGFR equations [28]. According to our research, it may be acceptable to choose an eGFR formula based on combined creatinine and cystatin C before a better formula appears. It is worth mentioning that, our reseach proves that, the novel FAS equations [11] are suitable for Chinese population, and even have superiority compared to other formulas in many cases, especially the eGFR_FAS_Cr_CysC_ equation. In a multicenter study of 1184 patients in China, the performance of the eGFR_FAS_Cr_CysC_ equation was better than that of the eGFR_EPI_Cr_CysC_2012_ equation, particularly in the elderly [29]. It may be necessary to further modify the FAS equation from a larger-scale study to make it more suitable for the Chinese population.

Exactly, each eGFR formula shows different clinical value in different BMI intervals. Therefore, the BMI of patients with CKD is an aspect worthy considering when choosing the appropriate eGFR equation. Which is the best choice? In our study, in normal or low-weight population, the formula based on serum creatinine is preferred, and in overweight or obese population, the formula based on serum creatinine and cystatin C may be more suitable. The reason may be that the combination of both biomarkers can cancel out the non-GFR-related factors influencing creatinine and cystatin C in different directions compared with mGFR. Steubl et al. suggest that combining metabolites or proteins in equations to minimize the influence of nonkidney-related parameters appears to be a promising approach which is consistent with our view [30].

Our research had some strengths such as on ethnic factors that all were from Chinese population, concentrative age range, common high-risk diseases for CKD. These favoured us to identify the appropriate eGFR equation for Chinese population while considering the impact of BMI. However, it needs to be verified and confirmed by different types of studies based on a larger population. More comparative studies on different types of samples are needed to further illuminate which biomarkers are better tools for diagnosis and prognosis of CKD.

Nevertheless, our study has some limitations. Firstly, we did not obtain specific data such as appendicular lean mass index (ALMI) and total body fat percentage (TBF%) measured by dual-energy x-ray absorptiometry (DXA) of body composition [31, 32], so we cannot refine the population in the BMI intervals. Secondly, we didn’t have enough data of proteinuria to define renal dysfunction because we only got one urine protein test result for each patient’s first morning urine. Thirdly, as it was a cross-sectional analysis, and a retrospective, single-center study, the results of this study should be carefully applied in practical clinical practice. Finally, although we assessed eight eGFR equations that were commonly used, there are also some other equations which were well praised were not included in our study.

In conclusion, after comprehensive analysis of factors that included consistency, accuracy, classification ability and diagnostic performance, we tend to suggest that choosing eGFR_EPI_Cr_2009_ or eGFR_FAS_Cr_ equation to estimate GFR of patients when BMI is around 20.9 kg/m^2^, eGFR_FAS_Cr_CysC_ for overweight patients (BMI around 24.8 kg/m^2^), and eGFR_EPI_Cr_CysC_2012_ for obese patients (BMI is about 28.9 kg/m^2^).

## Supplementary Information

**Additional file 1: Supplemental Table 1.** CKD stage classification based on eGFRs in BMI_P25_ interval. **Supplemental Table 2.** CKD stage classification based on eGFRs in BMI_P25–75_ interval. **Supplemental Table 3.** CKD stage classification based on eGFRs in BMI_P75_ interval. **Supplemental Table 4.** Diagnostic performance in BMI_P25_ interval of eGFRs for predicting renal insufficiency (mL/min/1.73 m2). **Supplemental Table 5.** Diagnostic performance in BMI_P25–75_ interval of eGFRs for predicting renal insufficiency (mL/min/1.73 m2). **Supplemental Table 6.** Diagnostic performance in BMI_P75_ interval of eGFRs for predicting renal insufficiency (mL/min/1.73 m2).

**Additional file 2.**

## Data Availability

All data analysed during this study are included in Supplementary Information Files and also available from the corresponding author on reasonable request.

## References

[1] Kidney Disease: Improving Global Outcomes (KDIGO) CKD Work Group (2013). KDIGO 2012 Clinical Practice Guideline for the Evaluation and Management of Chronic Kidney Disease. Kidney Int.

[2] Jha V, Garcia-Garcia G, Iseki K, Li Z, Naicker S, Plattner B, Saran R, Wang AY, Yang CW (2013). Chronic kidney disease: global dimension and perspectives. Lancet.

[3] GBD Chronic Kidney Disease Collaboration (2020). Global, regional, and national burden of chronic kidney disease, 1990-2017: a systematic analysis for the global burden of disease study 2017. Lancet..

[4] Trimarchi H, Muryan A, Martino D, Toscano A, Iriarte R, Campolo-Girard V, Forrester M, Pomeranz V, Fitzsimons C, Lombi F, Young P, Raña MS, Alonso M (2012). Creatinine- vs. cystatin C-based equations compared with 99mTcDTPA scintigraphy to assess glomerular filtration rate in chronic kidney disease. J Nephrol.

[5] Vinge E, Lindergard B, Nilsson-Ehle P, Grubb A (1999). Relationships among serum cystatin C, serum creatinine, lean tissue mass and glomerular filtration rate in healthy adults. Scand J Clin Lab Invest.

[6] Bouquegneau A, et al. Modification of Diet in Renal Disease versus Chronic Kidney Disease Epidemiology Collaboration equation to estimate glomerular filtration rate in obese patients. Nephrol Dial Transplant. 2013:28, iv122–iv130. 10.1093/ndt/gft329. 10.1093/ndt/gft329 24026245

[7] Lemoine S, Guebre-Egziabher F, Sens F, Nguyen-Tu MS, Juillard L, Dubourg L, Hadj-Aissa A (2014). Accuracy of GFR estimation in obese patients. Clin J Am Soc Nephrol.

[8] Wasén E, Isoaho R, Mattila K, Vahlberg T, Kivelä SL, Irjala K (2003). Serum cystatin C in the aged: relationships with health status. Am J Kidney Dis.

[9] Levey AS, Stevens LA, Schmid CH, Zhang Y(L), Castro AF, Feldman HI, Kusek JW, Eggers P, van Lente F, Greene T, Coresh J, for the CKD-EPI (Chronic Kidney Disease Epidemiology Collaboration) (2009). A new equation to estimate glomerular filtration rate. Ann Intern Med.

[10] Inker LA, Schmid CH, Tighiouart H, Eckfeldt JH, Feldman HI, Greene T, Kusek JW, Manzi J, Van Lente F, Zhang YL (2012). Estimating glomerular filtration rate from serum creatinine and cystatin C. N Engl J Med.

[11] Pottel H, Delanaye P, Schaeffner E, Dubourg L, Eriksen BO, Melsom T, Lamb EJ, Rule AD, Turner ST, Glassock RJ, de Souza V, Selistre L, Goffin K, Pauwels S, Mariat C, Flamant M, Ebert N (2017). Estimating glomerular filtration rate for the full age spectrum from serum creatinine and cystatin C. Nephrol Dial Transplant.

[12] McKillop DJ, Cairns B, Duly E, Van Drimmelen M, Ryan M (2006). The effect of serum creatinine method choice on estimated glomerular filtration rate determined by the abbreviated MDRD formula. Ann Clin Biochem.

[13] Ma YC, Zuo L, Chen JH, Luo Q, Yu XQ, Li Y, Xu JS, Huang SM, Wang LN, Huang W, Wang M, Xu GB, Wang HY (2006). Modified glomerular filtration rate estimating equation for Chinese patients with chronic kidney disease. J Am Soc Nephrol.

[14] Smirnov N (1948). Table for estimating the goodness of fit of empirical distributions. Ann Math Stat.

[15] Hart A (2001). Mann-Whitney test is not just a test of medians: differences in spread can be important. BMJ..

[16] Bland JM, Altman DG (1986). Statistical methods for assessing agreement between two methods of clinical measurement. Lancet..

[17] Chi XH, Li GP, Wang QS, Qi YS, Huang K, Zhang Q, Xue YM (2017). CKD-EPI creatinine-cystatin C glomerular filtration rate estimation equation seems more suitable for Chinese patients with chronic kidney disease than other equations. BMC Nephrol.

[18] Landis JR, Koch GG (1977). The measurement of observer agreement for categorical data. Biometrics..

[19] DeLong ER, DeLong DM, Clarke-Pearson DL (1988). Comparing the areas under two or more correlated receiver operating characteristic curves: a nonparametric approach. Biometrics..

[20] Sarnak MJ, Amann K (2019). Chronic Kidney Disease and Coronary Artery Disease: JACC State-of-the-Art Review. J Am Coll Cardiol.

[21] Lamb EJ, Tomson CR, Roderick PJ, clinical sciences reviews Committee of the Association for clinical biochemistry (2005). Estimating kidney function in adults using formulae. Ann Clin Biochem.

[22] Stenvinkel P, Zoccali C, Ikizler TA (2013). Obesity in CKD--what should nephrologists know?. J Am Soc Nephrol.

[23] Wang Y, Chen X, Song Y, Caballero B, Cheskin LJ (2008). Association between obesity and kidney disease: a systematic review and meta-analysis. Kidney Int.

[24] Levey AS, Inker LA (2017). Assessment of glomerular filtration rate in health and disease: a state of the art review. Clin Pharmacol Ther.

[25] Stevens LA, Schmid CH, Greene T, Li L, Beck GJ, Joffe MM, Froissart M, Kusek JW, Zhang YL, Coresh J, Levey AS (2009). Factors other than glomerular filtration rate affect serum cystatin C levels. Kidney Int.

[26] Teaford HR, Barreto JN, Vollmer KJ, Rule AD, Barreto EF, Cystatin C (2020). A Primer for Pharmacists. Pharmacy (Basel).

[27] Yang M, Xu G, Ling L, Niu J, Lu T, Du X, Gu Y (2017). Performance of the creatinine and cystatin C-based equations for estimation of GFR in Chinese patients with chronic kidney disease. Clin Exp Nephrol.

[28] Chang AR, Zafar W, Grams ME (2018). Kidney Function in Obesity-Challenges in Indexing and Estimation. Adv Chronic Kidney Dis.

[29] Yong Z, Li F, Pei X, Liu X, Song D, Zhang X, Zhao W (2019). A comparison between 2017 FAS and 2012 CKD-EPI equations: a multi-center validation study in Chinese adult population. Int Urol Nephrol.

[30] Steubl D, Inker LA (2018). How best to estimate glomerular filtration rate? Novel filtration markers and their application. Curr Opin Nephrol Hypertens.

[31] Thibault R, Pichard C (2012). The evaluation of body composition: a useful tool for clinical practice. Ann Nutr Metab.

[32] Marinangeli CP, Kassis AN (2013). Use of dual X-ray absorptiometry to measure body mass during short- to medium-term trials of nutrition and exercise interventions. Nutr Rev.

