# Supplementary material for: Choosing an appropriate glomerular filtration rate estimating equation: role of body mass index
Source: BMC Nephrol. 2021 May 25;22:197. doi: 10.1186/s12882-021-02395-x (PMC8145837; doi:10.1186/s12882-021-02395-x)
Supplement: Supplementary file 1 — Additional file 1: Supplemental Table 1. CKD stage classification based on eGFRs in BMIP25 interval. Supplemental Table 2. CKD stage classification based on eGFRs in BMIP25–75 interval. Supplemental Table 3. CKD stage classification based on eGFRs in BMIP75 interval. Supplemental Table 4. Diagnostic performance in BMIP25 interval of eGFRs for predicting renal insufficiency (mL/min/1.73 m2). Supplemental Table 5. Diagnostic performance in BMIP25–75 interval of eGFRs for predicting renal insufficiency (mL/min/1.73 m2). Supplemental Table 6. Diagnostic performance in BMIP75 interval of eGFRs for predicting renal insufficiency (mL/min/1.73 m2). [file 12882_2021_2395_MOESM1_ESM.docx]

Choosing an appropriate glomerular filtration rate estimating equation: Role of Body Mass Index

Jiayong Li^1#^, Xiang Xu^1#^, Jialing Luo^1^, Wenjing Chen^1^, Man Yang^2^, Ling Wang^2^, Nan Zhu^2^, Weijie Yuan^2^, Lijie Gu^2*^

1. Clinical Laboratory Medicine Center, Shanghai General Hospital, Shanghai Jiao Tong University School of Medicine, Shanghai, 200080, China

2. Department of Nephrology, Shanghai General Hospital, Shanghai Jiao Tong University School of Medicine, Shanghai, 200080, China

*Corresponding author. Gu Lijie, Email: [lijiegu82@126.com](mailto:ywj4169@163.com)

# The authors contributed equally to this work.

**Supplemental Table 1** CKD stage classification based on eGFRs in BMI_P25_ interval

| Equation | CKD stage based on mGFR | | | | | Kappa |
| --- | --- | --- | --- | --- | --- | --- |
|  | Stage 1 | Stage 2 | Stage 3 | Stage 4 | Stage 5 |  |
| eGFREPI_Cr_2009 | 65（84.4%） | 34（45.9%） | 19（45.2%） | 1（8.3%） | 4（100.0%） | 0.418 |
| eGFREPI_CysC_2012 | 37（48.1%） | 28（37.8%） | 24（57.1%） | 5（41.7%） | 4（100.0%） | 0.282 |
| eGFREPI_Cr_CysC_2012 | 31（40.3%） | 34（45.9%） | 21（50.0%） | 1（8.3%） | 4（100.0%） | 0.238 |
| eGFR_FAS_Cr_ | 59 (76.6%) | 32 (43.2%) | 25 (61.0%) | 4(30.8%) | 3(75.0 %) | 0.422 |
| eGFR_FAS_CysC_ | 21 (27.3%) | 33 (44.6%) | 28 (68.3%) | 8(61.5%) | 2(50.0%) | 0.239 |
| eGFR_FAS_Cr_CysC_ | 40 (51.9%) | 40 (54.1%) | 29 (70.7%) | 6(46.2%) | 3(75.0%) | 0.398 |
| eGFRa_MDRD | 65（84.4%） | 32（43.2%） | 18（42.9%） | 2（16.7%） | 4（100.0%） | 0.412 |
| eGFRc_MDRD | 72（93.5%） | 23（31.1%） | 21（50.0%） | 1（8.3%） | 4（100.0%） | 0.398 |

**Supplemental Table 2** CKD stage classification based on eGFRs in BMI_P25-75_ interval

| Equation | CKD stage based on mGFR | | | | | Kappa |
| --- | --- | --- | --- | --- | --- | --- |
|  | Stage 1 | Stage 2 | Stage 3 | Stage 4 | Stage 5 |  |
| eGFREPI_Cr_2009 | 92（86.8%） | 66（36.9%） | 50（50.0%） | 9（32.1%） | 6（100.0%） | 0.357 |
| eGFREPI_CysC_2012 | 57（53.8%） | 110（61.5%） | 61（61.0%） | 16（57.1%） | 6（100.0%） | 0.431 |
| eGFREPI_Cr_CysC_2012 | 52（49.1%） | 110（61.5%） | 64（64.0%） | 13（46.4%） | 6（100.0%） | 0.415 |
| eGFR_FAS_Cr_ | 88 (83.0%) | 72 (40.2%) | 58 (58.0 %) | 18(64.3%) | 6(100.0 %) | 0.415 |
| eGFR_FAS_CysC_ | 41 (38.7%) | 102 (57.0%) | 76 (76.0%) | 23(82.1%) | 0(00.0%) | 0.401 |
| eGFR_FAS_Cr_CysC_ | 66 (62.3%) | 114(63.7%) | 68 (68.0%) | 20(71.4%) | 5(83.3%) | 0.504 |
| eGFRa_MDRD | 89（84.0%） | 73（40.8%） | 51（51.0%） | 8（28.6%） | 6（100.0%） | 0.366 |
| eGFRc_MDRD | 97（91.5%） | 59（33.0%） | 49（49.0%） | 7（25.0%） | 6（100.0%） | 0.343 |

**Supplemental Table 3** CKD stage classification based on eGFRs in BMI_P75_ interval

| Equation | CKD stage based on mGFR | | | | | Kappa |
| --- | --- | --- | --- | --- | --- | --- |
|  | Stage 1 | Stage 2 | Stage 3 | Stage 4 | Stage 5 |  |
| eGFREPI_Cr_2009 | 35（92.1%） | 34（40.0 %） | 27（38.6%） | 5（35.7 %） | 1（50.0%） | 0.292 |
| eGFREPI_CysC_2012 | 22（57.9 %） | 42（49.4 %） | 49（70.0%） | 8（57.1%） | 2（100.0%） | 0.407 |
| eGFREPI_Cr_CysC_2012 | 23（60.5%） | 51（60.0%） | 50（71.4%） | 8（57.1%） | 2（100.0%） | 0.484 |
| eGFR_FAS_Cr_ | 34 (89.5%) | 38 (44.7%) | 36(51.4 %) | 7 (50.0%) | 1 (50.0 %) | 0.375 |
| eGFR_FAS_CysC_ | 16 (42.1%) | 44 (51.8%) | 56 (80.0%) | 7 (50.0%) | 0 (00.0%) | 0.392 |
| eGFR_FAS_Cr_CysC_ | 27 (71.1%) | 52 (61.2%) | 49 (70.0%) | 6 (42.9%) | 1 (50.0%) | 0.485 |
| eGFRa_MDRD | 34（89.5%） | 36（42.4%） | 33（47.1%） | 4（28.6%） | 2（100.0%） | 0.331 |
| eGFRc_MDRD | 37（97.4%） | 28（32.9%） | 26（37.1%） | 5（35.7%） | 1（50.0%） | 0.269 |

**Supplemental Table 4** Diagnostic performance in BMI_P25_ interval of eGFRs for predicting renal insufficiency (mL/min/1.73 m2)

| Equation | eGFR_EPI_Cr_2009_ | eGFR_EPI_CysC_2012_ | eGFR_EPI_Cr_CysC_2012_ | eGFR_FAS_Cr_ | eGFR_FAS_CysC_ | eGFR_FAS_Cr_CysC_ | eGFR_a_MDRD_ | eGFR_c_MDRD_ |
| --- | --- | --- | --- | --- | --- | --- | --- | --- |
| Cutoff Value | 67.1 | 50.6 | 48.7 | 56.6 | 46.8 | 53.7 | 64.5 | 77.1 |
| AUC_60_(95% CI) | 0.920  (0.847-0.953) | 0.911  (0.864-0.946) | 0.931  (0.887-0.961) | 0.928 (0.884-0.959) | 0.908^●^ (0.860-0.943) | 0.930 (0.887-0.961) | 0.916  (0.869-0.950) | 0.913  (0.866-0.948) |
| Youden index J | 0.738 | 0.655 | 0.74 | 0.728 | 0.681 | 0.740 | 0.703 | 0.687 |
| Sensitivity | 89.7 | 82.8 | 87.9 | 82.8 | 79.3 | 87.9 | 86.2 | 87.9 |
| Specificty | 84.1 | 82.8 | 86.1 | 90.1 | 88.7 | 86.1 | 84.1 | 80.8 |
| Adjustment Cutoff Value | 61.8 | 59.6 | 60.0 | 60.8 | 59.9 | 59.5 | 60.4 | 58.9 |
| Sensitivity | 77.6 | 91.4 | 91.4 | 84.5 | 91.4 | 91.4 | 79.3 | 75.9 |
| Specificty | 87.4 | 69.5 | 70.9 | 84.1 | 69.5 | 79.5 | 85.4 | 89.4 |

^●^P=0.036，compared with eGFR_FAS_Cr_CysC_；

**Supplemental Table 5** Diagnostic performance in BMI_P25-75_ interval of eGFRs for predicting renal insufficiency (mL/min/1.73 m2)

| Equation | eGFR_EPI_Cr_2009_ | eGFR_EPI_CysC_2012_ | eGFR_EPI_Cr_CysC_2012_ | eGFR_FAS_Cr_ | eGFR_FAS_CysC_ | eGFR_FAS_Cr_CysC_ | eGFR_a_MDRD_ | eGFRc__MDRD_ |
| --- | --- | --- | --- | --- | --- | --- | --- | --- |
| Cutoff Value | 67.1 | 53.8 | 57.2 | 64.9 | 55.6 | 62.9 | 64.1 | 71.3 |
| AUC_60_(95%CI) | 0.929  (0.901-0.952) | 0.928  (0.899-0.951) | 0.939  (0.912-0.960) | 0.934 (0.905-0.956) | 0.928^●^ (0.899-0.951) | 0.941 (0.914-0.962) | 0.922  (0.892-0.946) | 0.921  (0.891-0.945) |
| Youden index J | 0.692 | 0.696 | 0.709 | 0.704 | 0.721 | 0.711 | 0.696 | 0.688 |
| Sensitivity | 82.8 | 83.6 | 88.1 | 86.6 | 91.0 | 92.5 | 83.6 | 82.1 |
| Specificty | 86.3 | 86.0 | 82.8 | 83.9 | 81.1 | 78.6 | 86.0 | 86.7 |
| Adjustment Cutoff Value | 60.0 | 60.1 | 60.2 | 60.1 | 60.1 | 60.0 | 60.0 | 59.8 |
| Sensitivity | 74.5 | 90.3 | 91.8 | 78.4 | 94.0 | 87.3 | 75.4 | 70.9 |
| Specificty | 90.5 | 78.9 | 77.2 | 86.7 | 70.9 | 82.5 | 89.8 | 92.3 |

^●^P=0.021，compared with eGFR_FAS_Cr_CysC_；

**Supplemental Table 6** Diagnostic performance in BMI_P75_ interval of eGFRs for predicting renal insufficiency (mL/min/1.73 m2)

| Equation | eGFR_EPI_Cr_2009_ | eGFR_EPI_CysC_2012_ | eGFR_EPI_Cr_CysC_2012_ | eGFR_FAS_Cr_ | eGFR_FAS_CysC_ | eGFR_FAS_Cr_CysC_ | eGFRa__MDRD_ | eGFR_c_MDRD_ |
| --- | --- | --- | --- | --- | --- | --- | --- | --- |
| Cutoff Value | 74 | 51.6 | 60.5 | 74.9 | 58.6 | 61.5 | 70.3 | 84.1 |
| AUC_60_ (95%CI) | 0.906  (0.858-0.942) | 0.900*  (0.851-0.937) | 0.919  (0.873-0.952) | 0.915 (0.869-0.949) | 0.904^●^ (0.856-0.940) | 0.922 (0.877-0.955) | 0.906  (0.858-0.942) | 0.905  (0.857-0.941) |
| Youden index J | 0.706 | 0.639 | 0.712 | 0.671 | 0.651 | 0.718 | 0.683 | 0.629 |
| Sensitivity | 86 | 74.4 | 90.7 | 90.7 | 89.5 | 87.2 | 83.7 | 84.9 |
| Specificty | 84.5 | 89.4 | 80.5 | 76.4 | 75.6 | 84.6 | 84.6 | 78.1 |
| Adjustment Cutoff Value | 60 | 60.4 | 59.9 | 60.1 | 60.0 | 59.7 | 59.0 | 58.7 |
| Sensitivity | 53.5 | 83.7 | 88.4 | 61.3 | 90.7 | 79.1 | 61.6 | 50.0 |
| Specificty | 92.7 | 77.2 | 81.3 | 91.9 | 74.0 | 87.0 | 91.9 | 95.1 |

*P < 0.05 compared with eGFR_EPI_Cr_CysC_2012_

^●^P=0.018，compared with eGFR_FAS_Cr_CysC_；
